# New Insights in Topical Drug Delivery for Skin Disorders: From a Nanotechnological Perspective

**DOI:** 10.1021/acsomega.2c08016

**Published:** 2023-05-19

**Authors:** Neha Raina, Radha Rani, Vijay Kumar Thakur, Madhu Gupta

**Affiliations:** † Department of Pharmaceutics, Delhi Pharmaceutical Sciences and Research University, Pushp Vihar, New Delhi 110017, India; ‡ Biorefining and Advanced Materials Research Center, SRUC (Scotland’s Rural College), Kings Buildings, West Mains Road, Edinburgh EH9 3JG, U.K.; § School of Engineering, University of Petroleum & Energy Studies (UPES), Dehradun 248007, Uttarakhand, India

## Abstract

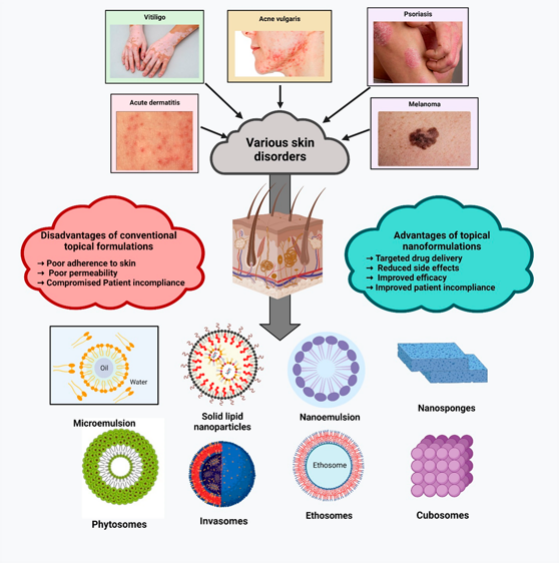

Skin, the largest organ in humans, is an efficient route for the delivery of drugs as it circumvents several disadvantages of the oral and parenteral routes. These advantages of skin have fascinated researchers in recent decades. Drug delivery via a topical route includes moving the drug from a topical product to a locally targeted region with dermal circulation throughout the body and deeper tissues. Still, due to the skin’s barrier function, delivery through the skin can be difficult. Drug delivery to the skin using conventional formulations with micronized active components, for instance, lotions, gels, ointments, and creams, results in poor penetration. The use of nanoparticulate carriers is one of the promising strategies, as it provides efficient delivery of drugs through the skin and overcomes the disadvantage of traditional formulations. Nanoformulations with smaller particle sizes contribute to improved permeability of therapeutic agents, targeting, stability, and retention, making nanoformulations ideal for drug delivery through a topical route. Achieving sustained release and preserving a localized effect utilizing nanocarriers can result in the effective treatment of numerous infections or skin disorders. This article aims to evaluate and discuss the most recent developments of nanocarriers as therapeutic agent vehicles for skin conditions with patent technology and a market overview that will give future directions for research. As topical drug delivery systems have shown great preclinical results for skin problems, for future research directions, we anticipate including in-depth studies of nanocarrier behavior in various customized treatments to take into account the phenotypic variability of the disease.

## Introduction

An effective route for drug delivery is the skin which circumvents many drawbacks of the oral, inhalation, and parenteral routes. Because of these skin benefits, it has intrigued investigators in the past years.^1^ The skin controls the entry and exit of many chemicals, preventing moisture loss and controlling body temperature to preserve balance as homeostasis within the body.^2,3^ Nearly one-third of the world’s population is affected by skin disorders, which are the fourth most common cause of all human diseases. Despite this, their impact is frequently underestimated. The high frequency of skin conditions, long-term morbidity, and the disability-adjusted life which includes severe itching, as in the case of atopic dermatitis and chronic inflammatory skin conditions like psoriasis, and the expensive cost of novel treatments like biologics all contribute to the burden of skin illnesses.^4^ Some healthcare systems may be economically threatened by the high incidence of skin cancer and the associated treatment expenses. The most significant burden among skin disorders is caused by atopic dermatitis, which is ranked 15th among all nonfatal diseases. Acne is a relatively common inflammatory dermatosis that is more prevalent in women and teenagers. Psoriasis is estimated to affect about 2–4% of the population in western countries.^2^ So, the treatment of these diseases by the topical route is effective, but for this a thorough understanding of skin as a barrier is essential. Skin is a complex barrier with three layers: the epidermis (outermost layer), the middle layer dermis (contains various connective fibers, sensory receptors, and sweat glands), and the hypodermis, which is the subcutaneous layer (has adipose tissue and anchors the other two skin layers for support).^5^ This route has been explored to a great extent for the delivery of drugs because of its user friendliness and larger surface area.^6,7^ The main route leading to these living layers of the skin is winding and highly hydrophobic. Therefore, drugs that successfully diffuse across the stratum corneum should be relatively smaller in size, lipophilic or amphiphilic in nature, and nonirritating. However, many potentially valuable drug and cosmetic compounds have properties that do not meet these requirements.^8^ To overcome these obstacles, attention has been focused not only on the active ingredient but also on the form and composition of the entire formulation of a delivery system. These benefits entice pharmaceutical firms to create topical treatments for skin conditions.^3,8^ The skin’s ability to act as a barrier reduces the efficacy of treatments using simple topical formulations such as lotions and creams.^6^ Numerous strategies have been investigated up to this point to get beyond the skin’s natural barriers and deliver drugs effectively. Currently, more and more percutaneous therapies use various types of nanometric scale transporters. Nanocarriers as drug carriers can potentially enhance drugs’ specificity, bioavailability, and therapeutic efficacy while improving patient compliance during therapy. Besides, nanoparticle-based drug delivery can enhance drug retention with tunable release kinetics at the disease site inside the skin.^9^ Skin entry of nanoparticles through the transappendageal route, which includes the hair follicles,^10^ sweat glands, and the sebaceous and pilosebaceous glands, has been reported.^11,12^ This enables nanoparticles to penetrate the superficial layers of the stratum corneum, i.e., the outermost protective layer of the skin.

However, the transappendageal route covers only 0.1% of the total skin surface.^13^ Consequently, it does not contribute significantly to the penetration of large molecules and nanoparticles into deeper layers of the skin where the disease is primarily localized. The applications of such novel nanovehicle systems can deliver potent drugs to the preferred site in an exact manner. The skin reservoirs created from designed nanosystems effectively control therapeutic agent release to the damaged area at the skin site with a localized effect. Moreover, the site-specific dermal targeting was facilitated by nanosized particles, and their narrow size distribution may increase medication retention.^14−16^ Novel approaches to skin delivery of bioactive substances may be based on the entrapment of therapeutically active agents in nanocarriers, which are progressively used in skin targeting and topical delivery. Such delivery vehicles aid release in a sustained manner, leading to prolonged activity or improved absorption and perhaps a reduction in deleterious effects.^17,18^ The prime goal of this current review is to assess the various innovative nanocarrier-based delivery systems employed to enhance therapeutic moiety uptake through the skin and their potential for treating disorders associated with the skin. Patents on topical delivery are mentioned with a brief overview of marketed topical products.

## History of Topical Products

The skin has been widely used for hundreds of years to deliver poorly soluble and low bioavailable drugs (when administered orally) worldwide. Africans from ancient times treated dermal disorders using phytochemicals, minerals, and cosmetic products like red ochers, kohl, and henna in 4000 BC. In 1500 BC, Ebers Papyrus wrote a book based on papyrus paper containing information like the use of the tiger nut for wound healing management.^19−22^ The emulsion-based cold cream of beeswax, water, and vegetable oil was first formulated by a Greek physician Galen for skin care after 1500 BC. These creams possessed good activity against microbial infections and so were used for treating burns and wounds in Greece and were further employed for the administration of mixed herbs as plasters and bandages by Chinese people in the ancient period. The mixed herbs and unprocessed rubber gums were applied as skin plaster for local treatment. Mercury ointment, named Unguentum Hydrargyriwas, was the first transcutaneous formulation developed to cure syphilis in the 15th century. After many years, in 1880, a German pharmacist formulated plaster “gutta-percha plaster gauze” for skin problems.^23−28^ In China, plasters with medical effects containing different herbs and drugs, for instance, sesame seed, castor oil, and moringa as protectants, diaphoretics, and astringent, were used in 2000 BCE.^29^ Historically, plasters have been widely employed for skin disorders. Still, drug delivery through transdermal and topical routes fetched the attention of researchers in the 20th century when phenol skin poisoning due to split off from plasters was studied.^30^ In 1896, a German physician specializing in dermatology published a histopathology of skin diseases in which he considered the skin as an organ for the delivery of dermatological therapeutics and defined the importance of topical treatment of skin problems.^31^ In the middle ages and ancient eras, several minerals, plant extracts, and dyes were applied topically that showed therapeutic activity with toxic effects because of the unawareness of the safety profile of the therapeutic agent. These products include blue-gray lead sulfide (Kohl) for antimicrobial activity from the time of Egyptians, silver metallic mercury (quicksilver), and decorative orange to red mercuric chloride (cinnabar). Initially, quicksilver ointment was used in Arab countries for skin problems. After that, Paracelsus, a Swiss alchemist and physician, added calomel, sublimate, and some other oxides or metallic salts to the quicksilver ointment for the treatment of syphilis in the European Renaissance. Paracelsus first used a topical mercury ointment to solve the toxicity of the mercury ointment under oral administration and became the establisher of toxicology of the modern era.^32^ Later on, in the 20th century, the topical use of mercury ointment for skin problems was replaced with the administration of penicillin. However, the use of mercury, along with phenylmercuric nitrate and phenyl mercuric acetate salts, was continued as a preservative added in topical formulations, particularly ophthalmic preparations.^33^ MercurochromeR, also known as merbromin, is a salt compound of organomercuric disodium. It did not contain any heavy metal and was widely applied topically for its antiseptic action, but it was still found to be toxic. Other topical formulations seen to possess adverse effects were poisoning from belladonna plaster, lotion, and liniments; headaches from nitroglycerin when dermal exposure occurred in explosive factories; and the toxicity of hexachlorophene to babies on topical application.^34,35^ Due to the adverse effects caused by therapeutic agents, vigilance was required. Vigilance is still needed to avoid systemic adverse effects of steroid suppression on topical application of glucocorticosteroid on damaged skin when used for a longer period and in young children. Several drugs applied to the skin cause adverse effects limited to layers of skin only, for instance, the effects of xenobiotics and corticosteroids that occasionally become serious when used for prolonged periods for striae, atrophy, acne, perioral dermatitis, purpura, and rosacea. These adverse effects have been caused by the vehicle used in the formulation, the site of topical application, and the chemistry of the steroids.^36^ With time, continuous technological advancements have increased the knowledge about the percutaneous absorption mechanism of drugs and improved the quality of advanced topical formulations. The understanding of skin morphology, pharmacology, toxicology, physiology, and pharmaceutical technology was enhanced because of a research explosion in the 20th and 21st centuries that included the quantification of drug permeation from topically applied drug product capable of penetrating the epidermis, human stratum corneum, and dermatomed skin.^37−39^ Conventionally, topical ointment and creams were used to treat skin diseases. Along with toxicity and adverse effects, some formulation-related problems also create limitations in the topical treatment. The drug from these formulations releases rapidly and makes a layer of concentrated drug and causes a greasy and sticky ointment, low efficiency of the carrier system, an unpleasant order, and uncontrolled evaporation of volatile substances from formulations. The invention of novel drug delivery systems overcomes the problems related to conventional topical formulations with the potential of enhanced therapeutic efficacy and reduced side effects and adverse effects.^40^ Liposomes were discovered in the year 1965 by Bangham and investigated continuously as the most efficient carrier system for biologically active compounds and drugs.^41^ These are spherical, vesicular drug delivery systems used widely and extensively for topical application. The econazole-loaded antifungal liposomal gel was the first topical liposome formulation designed in Switzerland in 1994.^42^ These spherical vesicles are identical with biological membranes and capable of loading hydrophilic and hydrophobic drugs. After liposomes, solid lipid nanoparticles (SLNs) are regarded as the next generation of delivery systems. The introduction of SLNs as a substitute for conventional colloidal carriers, such as emulsions, liposomes, and polymeric micro- and nanoparticles, is due to their small particle size and adhesive qualities similar to liposomes. They are made of well-tolerated excipients and can form films on the skin. The SLN’s distinct benefits are its reliability.^43^ In the year 1980, Mezei and Gulasekharam made an important contribution to scientific studies on the topical therapy of triamcinolone liposomes that provided an increased concentration of loaded drug in the layers between the dermis and epidermis and reduced the amount of drug in the systemic circulation as compared to conventional topical formulations of triamcinolone acetonide.^44^ Transport studies of liposomes *in vivo* and *in vitro* conditions established the accumulation of tretinoin and fluconazole, according to reports.^40^ After the updated review of El Maghraby et al. published in 2006, the interest of researchers focused toward the topical application of liposomes in different areas, especially to treat hair problems related to the sebaceous gland.^45^ Phospholipids in these vesicular systems are associated with an edge activator that destabilizes and deforms the lipid bilayer of liposomes. The edge activator used in these deformable carrier systems includes dipotassium glycyrrhizinate, tween 80, span 80, and sodium cholate.^46^ Ethosomes are another advancement in vesicular drug delivery systems that provides enhanced penetration and delivery of drugs to the deep layers of skin in the wide area of dermatological disorders. Touitou and his team in 2000 designed ethosomes containing ethanol in place of cholesterol that act as penetration enhancers with increased drug concentration between skin layers compared to conventional liposomal formulations.^47^ Ethosomes are successfully employed to treat various dermal disorders with enhanced penetration and increased transport of drugs within deeper dermal layers.^48^ The incorporation of additional penetration enhancers in the liposome formulation is another approach for the greater improvement of drug delivery in skin layers. The penetration enhancers used in liposomes include Transcutol (2-(2-ethoxy ethoxy) ethanol), cineole, and Labrador (capryl-caproyl macrogol 8-glyceride). These penetration enhancers significantly improve minoxidil delivery between skin layers compared to conventional and ethanol-containing liposomal formulations.^49^ Different types of vesicular drug delivery systems operated differently according to their composition as per the investigations. So, a combinational approach of deformable transferases and ethosomes is desirable to develop an elastic drug delivery system for penetration across skin layers that more efficiently cross the skin membrane barriers.^50^ Despite the vesicular drug carriers, some other lipid nanocarriers like lipidic nanoparticles and nanoparticles having diverse compositions have been recently employed as drug delivery vehicles and been proven to be a miracle in the topical delivery of several drugs for dermatological and also for cosmeceutical problems.

## Skin Target Sites and Barriers in Skin Epidermis

**Figure 1:**
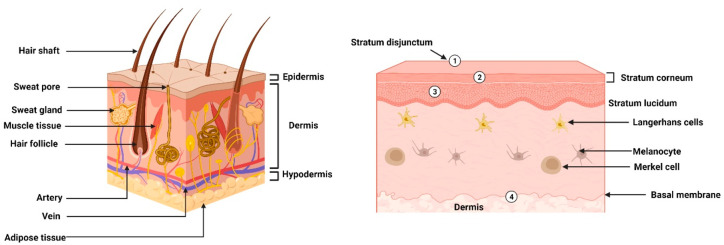
Morphology and physical barriers in the epidermis layers of skin. (1) Stratum disjunctum acts as a physical barrier that sheds and reforms at fixed intervals. (2) Stratum corneum lipids having mortar-like structure along with protein bricks make envelopes covering corneocytes, acting as physical barriers. (3) Denser and packed zones of the stratum corneum layer playing the role of a physical barrier. (4) Basal membrane at the interface of the epidermal and dermal layer that also behaves as a barrier for drug absorption.

Topical drug formulations are designed generally by keeping in mind that the drug is potent sufficiently for the targeted local action site. Various dermal, epidermal, and appendageal target site ranges of the skin (Figure 1) include Langerhans cells, Merkel cells, blood vessels, melanocytes, keratinocytes, and deeper tissues like muscle. The local therapeutic efficacy and adverse effects on systemic circulation should be considered to be balanced for the development of a topical formulation loaded with any potential drug. Locally used drugs for various therapeutic activities comprise melanocytes targeting skin-whitening agents, corticosteroids modifying keratinocyte and blood vessel behavior, topical anesthetics to lower local pain and itching due to the skin nervous system, anti-infective agents applied topically on the skin surface, and vaccine activation of Langerhans cells and retinoids.^31^ Skin plays important roles, like protection from the external environment, maintaining body temperature, and loss of water content from the body. Skin morphology and physical barriers to the absorption of topical products generally exist in the stratum corneum (SC) layer of skin. In addition to the nonviable physical barriers, i.e., the SC layer, epidermis, and dermis are other important viable physical barriers present in the skin. Brody categorizes the SC layer into three zones: (i) the basal zone, a densely packed zone of 4–10 keratin fibrils; (ii) the intermediate zone, denser than the basal zone comprising 8–12 loosely packed keratin cells; and (iii) the superficial zoneless dense zone having 2–3 keratin cells and space opacity between cells.^51^ Flat hexagonal-shaped corneocytes (anucleated cells of keratinocyte lineage) are 40 mm in diameter, up to 1 mm wide, irregular, and parallel to the surface of the skin, making the outermost layer of the epidermis.^52^ The superficial zone is the outermost layer of SC, also called stratum disjunction, that acts as a skin barrier, shedded and reformed at continuous intervals, for instance, 21 days for the back of the hands and 7 days for the forehead.^53^ Drug delivery depends upon the interactions between topical formulations and the dynamic barrier SC. Tight joints between corneocytes in the lower portion of the stratum basale and hook-shaped overlapping end sometimes retard or prolong the interval of stratum disjunction shedding.^54^ Water is the important component of topical carriers that causes a modulation in the SC layer, and the egression of water from the skin can be prevented by applying moisturizers. Compared to dry SC, hydrated, softened, and thick SC reduced the undulation of corneocytes.^31^ Richter and his team characterized changes in the thickness of three zones after hydration induced with the help of osmosis. One to three layers of corneocyte thickness decreased in the first zone.^55^ The second zone’s five to ten layers of corneocytes remain unaffected by osmotic effects. The changes to zone 1 were more than in zone 3.^56^ As per studies, only the water content present in stratum disjunction is changed due to SC hydration by topical formulation. No change was found in the water content of the stratum compactum. Hydration of SC also affects the protease activity of the protective and insoluble corneocyte envelope. The corneocyte envelopes are present at two sites with two different forms, i.e., at the lower side in SC layers, occurring as a deformable, irregular in shape, and fragile envelope, and at superficial SC layers, as a resilient and polygon-shaped envelope.^57^ Proteins in the SC layer are cross-linked with each other by disulfide bonds making protein bricks and lipids making a mortar-like assembly.^58^ These protein bricks provide biochemical strength to corneocytes by surrounding them, and the mortar structure of lipids acts as a functional barrier.^59^ For absorption by the dermal route, the drug should be capable of permeating the functional barrier of SC lipids and reaching the corneocyte brick. Whenever the drug cannot enter the corneocytes due to the surrounding protein brick, the drug particle binds with the corneodesmosomes and remains in the binding form with tight junctions at the edges of corneocytes. This type of corneocyte binding generally occurs in the deeper interfacing areas of the stratum granulosum of the SC layer.^60^ Along with keratinocytes, other types of cells are also present in the epidermal layer, like melanocytes, Merkel cells, and Langerhans. Melanocytes are dopapositive cells that originate from the neural crest, function to secrete melanin, and are stored in the melanosomes.^61^ Like melanocytes, some other negative cells are also present in the epidermis named Langerhans cells, but these cells do not secrete any pigment. These cells are named after the scientist Langerhans, who first identified these cells.^62,63^ Langerhans cells of an epidermal layer formed in the bone marrow and then were transported to the epidermis through the blood vessel walls of the dermis.^64^ Langerhans cells of the epidermis, after taking skin antigens, migrate to lymph nodes of that local region of the skin and activate T-cells, hence acting as antigen-presenting cells that help the body fight skin infections. Merkel cells are also known as Tastzellen or touch cells because they are present in touch-sensitive areas of the basal epidermal layer.^65^ The basement membrane forming the interface between the epidermis and dermis is another filter and barrier in skin morphology for migration, adhesion, differentiation, and anchorage of cells. Apart from the major function, i.e., supporting the adherence of the epidermis and dermis, this membrane also serves as a barrier for the movement of certain molecules and cells across this interface.^66^ However, skin morphology is heterogeneous at various body sites, having a different impact on drug absorption from topical formulations. Still, topical delivery of a drug is considered a substitute for the systemically administered drugs used for local infections and other disorders of the skin. Drug delivery by the topical route bypasses the problems of oral administrations like pH change and interference of food present in the gastrointestinal tract and the first-pass hepatic metabolism and increases the bioavailability of drugs that undergo biotransformation. The topical route is the preferred route for the administration of drugs having a short biological half-life and controlled drug release and also provides patient compliance with reduced dosing frequency. The topical route has also become a constraint in some cases; for instance, drugs with particle sizes more than 500 Da^67^ are not capable of crossing skin barriers present in the SC layer.^68,69^ All drugs are not ideal for loading in topical formulations for skin delivery. Only the drugs capable of penetrating the skin layer and delivering to a target site within the skin are selected for topical delivery to meet the need and perceptions of consumers.^70^ Despite all of this, the physicochemical profile of drugs and vehicles, dosing conditions, and skin health are some other factors that resist drug delivery through topical drug delivery.^71^

## Treatment Approaches for Various Disorders Using Topical Drug Delivery

**Figure 2:**
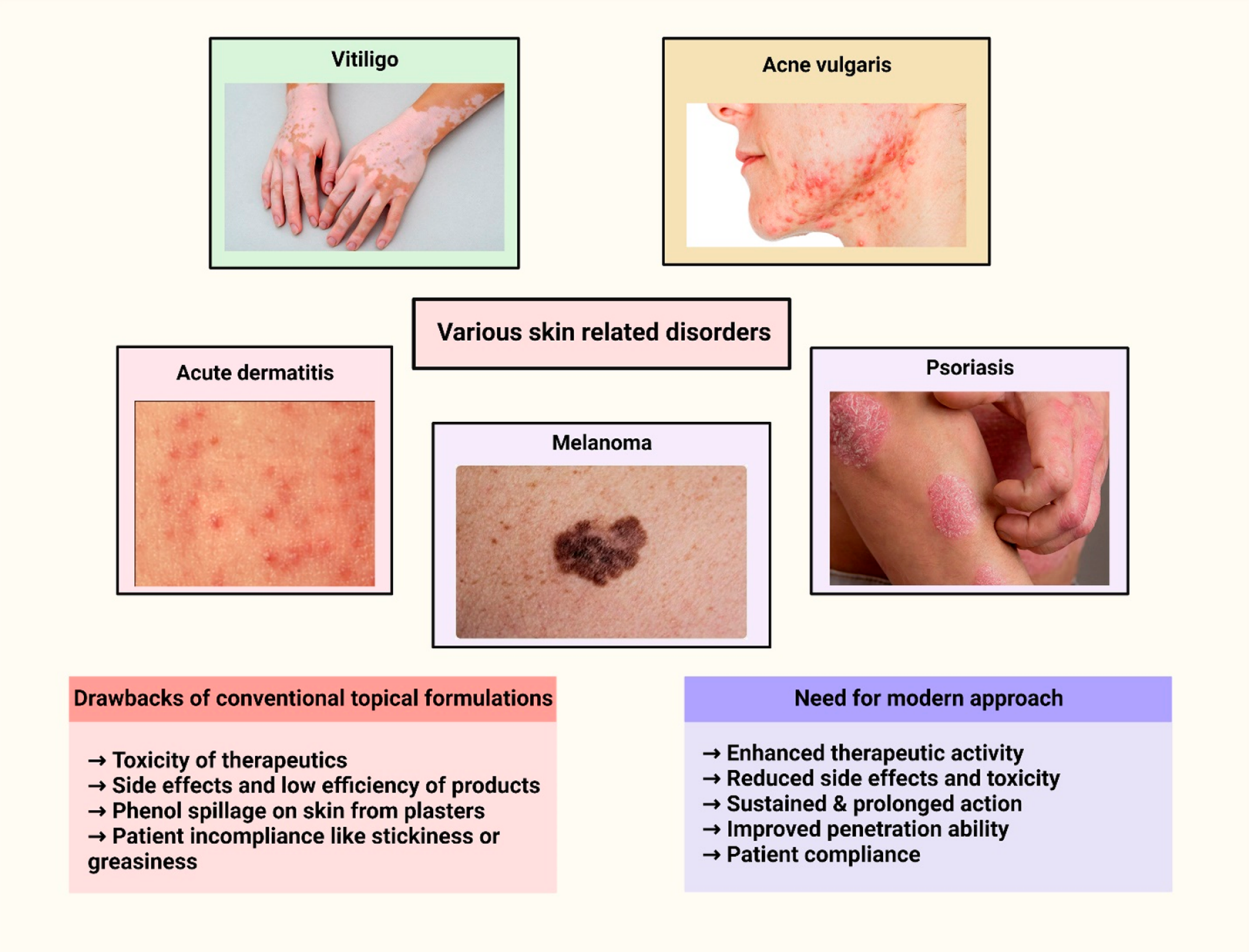
Treatment of skin disorders using topical therapy.

The skin controls the entry and exit of many chemicals, preventing moisture loss and controlling body temperature to preserve balance as homeostasis within the body. Topical medication delivery systems (Table1) obviously depend on the drug being able to transmit into the skin’s barrier and reach its intended delivery site.^72,73^ Various skin disorders (Figure 2) treated by the topical delivery approach are mentioned below.

**Table 1:** Topical Drug Delivery Intended for Several Skin Diseases

Disease	Therapeutic agent	Animal model	Route	Reference
Acne	Adapalene	Rabbit auricle	Topical	(74)
Acne vulgaris	Retinyl palmitate	Female Wistar rats	Topical	(75)
Acne	Azelaic acid, tea tree oil	Female Wistar rats, testosterone-induced skin acne male Swiss albino mice	Topical	(76)
Acne	Dapsone	Male BALB/c mouse	Topical	(77)
Acne	Isotretinoin, clindamycin phosphate	Testosterone-induced skin acne male laca mice	Topical	(78)
Skin fungal infections	Ketoconazole	Albino rabbits, Wistar rats	Topical	(79)
Skin infections and disorders	Rhein	Male Wistar rats	Topical	(80)
Skin infection	Garvicin KS, micrococcin P1	BALB/cJRj mice	Topical	(81)
Skin fungal infections	Miconazole	Albino rats	Topical	(82)
Skin fungal infections	Luliconazole	*Candida albicans* induced skin fungal infection Albino rats	Topical	(83)
Psoriasis	Gemcitabine HCL, tacrolimus, methotrexate sodium, triamcinolone, betamethasone 17-valerate	12-*O*-Tetradecanoylphorbol 13-acetate-induced skin hyperplasia and inflammation male Swiss mice	Topical	(84)
Face skin cancer	Small interfering RAN	Mouse xenograft model	Topical	(85)
Inflammatory skin disorders	Pioglitazone	Arachidonic-induced inflammatory skin BALB/c male mice	Topical	(86)
Psoriatic skin lesions	Imiquimod, curcumin	Psoriasis-induced mice, skin of male Albino rats	Topical	(87)
Cutaneous leishmaniasis	Amphotericin B	Female BALB/c mice	Topical	(88)

### Acne

The skin condition that affects a person’s appearance and is accompanied by chronic inflammation of the sebaceous glands, usually in the back and face area, is termed acne.^89,90^ The factors responsible for acne development are, namely, colonization of propionibacterium acnes, excessive androgen levels, proinflammatory cytokine release, and abnormal keratinization of the sebaceous glands.^91−93^ The liposomal hydrogel (3DP-NH), using reverse phase evaporation techniques and containing Cryptotanshinone (CPT), was formulated for pimples. Three-dimensional (3D) printing technologies have the ability to facilitate the personalized treatment of acne. The *in vitro* evaluation depicted that the size range of CPT-loaded niosomes was less than 150 nm, having an efficiency of entrapment between 67 and 71%. *In vivo* evaluation using an acne rat model for antiacne action demonstrated that 3DP-CPT-NH exerted a greater antiacne effect without skin irritation, improved hydration of the skin, and widened gaps of intercorneocyte in the stratum corneum. It can be concluded that for personalized acne treatment 3DP-CPT-NH is a promising topical drug delivery system.^94^ Alam et al. prepared a hydrogel composed of erythromycin estolate and isotretinoin-encapsulated microemulsion for acne treatment. The developed microemulsion-based hydrogel system, as compared to conventional products, exhibited increased permeation efficiency and more deposition across the layers of rodent skin for encapsulated drugs and enhanced the *in vitro* efficacy against *C. acnes*. Therefore, the formulated system could be a promising carrier for the topical application of both encapsulated therapeutic agents along with management of acne in a simple, effective, and safer approach.^95^ Kashani-Asadi-Jafari et al. formulated topical liposomes encapsulated with doxycycline hyclate for the treatment of acne and also to prevent the side effects of loaded drugs on oral administration. The prepared drug-loaded formulation exhibited increased activity against the main acne-causing bacterial strain and improved cell viability with almost three times more deposition in dermal layers of rat skin and nontoxicity to human dermal fibroblasts relative to free drug.^96^ Ethosomal gel using Carbopol and entrapping karanjin was prepared by Ansari et al. for effective skin acne therapy and improved delivery of the drug by a topical route. The developed gel was a nonirritant and exhibited an enhanced permeation rate to rat skin during histopathological evaluation and the Draize score test. Also, the bacterial colony zones of *Staphylococcus epidermidis* and *Propionibacterium acnes* were inhibited, along with lesser and smaller dermal units of sebaceous gland action observed. Using ethosomal gels as the potentially effective carrier system, there is a therapeutic possibility to enhance the topical distribution of karanjin in the treatment of acne.^97^ Codelivery approaches of loading azelaic acid and tea tree oil within an ethosome through a solvent injection technique were performed for acne treatment. Further, Carbopol was incorporated to form a hydrogel of drug-loaded ethosome and compared with marketed products. The prepared hydrogel exhibited improved drug permeation and retention time across dermal layers of the Wistar rat model, along with a safety profile.^98^ Conventional formulations are not significant for the acne caused by *Propionibacterium acnes* due to the limitation of the biofilm formation treatment. This study depicted that ethosomes for the photodynamic therapeutic potential of loaded hexyl-aminolevulinate (HAL) were evaluated against inflammatory Sprague–Dawley rats *in vivo* acne model and *in vitro* biofilm profiles of *P. acnes*. The prepared HAL ethosomes showed significant inhibition of biofilm formation as compared to plain HAL solution, suggesting the potential application of these ethosomes against biofilm formation in acne management.^99^

### Atopic Dermatitis

Atopic dermatitis (AD) or eczema is a skin condition with chronic inflammation that exhibits signs of extreme swelling, oozing in patients, itching, and redness.^100−102^ The current investigation for this disorder involves the use of Cephalosporin A (C_S_A) nanocapsules (NCs) topically. Drug penetration was enhanced in the several layers of porcine ear skin with the CsA-NCs. In terms of better protection of the integrity of the skin barrier, a decline in systemic pro-inflammation markers and decreased skin inflammation were seen with the use of the topical formulation of CsA-NCs. Compared with the current topical therapeutic drugs in AD, the overall experimental findings indicate that this novel topical platform can provide better efficacy in AD treatment.^103^ Kildaci et al. formulated and performed an *in vitro* and *in silico* evaluation of nanoemulsions encapsulated with linseed oil (LSO) by the technique of ultrasonic emulsification for the treatment of AD. The *in vitro* profile of LSO-NE resulted in nontoxicity to the Salmonella/Ames assay strain. From the findings, it was suggested that these LSO-NEs possessed high cellular and dermal permeability. Also, the topical application of these formulations could be proved as a potential therapy in the cure of AD.^104^ Almawash et al. coloaded levocetirizine dihydrochloride and fluticasone propionate in a microemulsion formulation for AD treatment. From the *in vivo* evaluation carried out on the rodent model, it was found that this system provided enhanced efficacy and release of encapsulated drugs at a controlled rate and was also nontoxic during the histopathological examination. Hence, it could be a simple and manageable strategy for the cure of AD.^105^ Tea tree oil (TTO) encapsulated ethosomal cream was formulated and evaluated with a comparison of the marketed cream of TTO by Kumar and co-workers to permeate and deposit a higher concentration in skin layers for managing AD. Ethanol and phosphatidylcholine were used as penetration enhancers and lipids to form ethosomes. The HaCaT skin cell lines and BALB/c mice were used for *ex vivo* and *in vivo* studies, respectively. From the findings, it was observed that the prepared ethosomal cream deposited more in skin layers, was safe during cell line examination, and efficiently minimized inflammation through IgE antibodies, WBC, eosinophils infiltration, and a decreased clinical score as compared to a simple TTO cream. Hence, it has been approved as more efficacious for AD treatment.^106^ Three vesicular cyanocobalamin-loaded formulations, i.e., ethosomes, liposomes, and transferosomes, through a film-hydration technique were prepared with better permeation efficiency across skin layers for the scavenging activity against nitric oxide in pathological conditions of AD and psoriasis. The prepared lipid vesicles exhibited higher penetration across deeper skin layers of porcine and increased systemic concentration of cyanocobalamin. So, the present study suggested lipid vesicles as a potential carrier system for the topical application of cyanocobalamin in psoriasis and AD treatment.^107^

### Melanoma

The deadliest type of skin cancer is melanoma, and its successful treatment is possible in the initial stage with the help of surgery alone and has a higher rate of survival. However, the survival rates decrease considerably after metastasis.^108,109^ Therefore, the topical application of chemotherapy is an effective route for successful skin cancer treatment.^110,111^ The chitosan-based 5-fluorouracil (FU) loaded pH-responsive biodegradable nanogel (FCNGL) was fabricated through the ion gelation technique for melanoma treatment. The formulation was effective against melanoma at even the lowest concentration (0.2% w/v) and showed selective drug accumulation at the melanoma site. FCNGL was successful in MTT and apoptosis experiments. The examination of the tumor using immunohistochemistry (IHC) showed that the subcutaneous layer symmetry and the layer of epithelial skin improved. The results clearly showed that for topical chemotherapy FCNGL has the potential for effective delivery of 5-FU in a sustained manner.^112^ Tang et al. studied the activity of tetrahydrocurcumin (THC) in the B16F10 cell model for melanoma. Results showed that THC significantly reduced melanin production by constraining the α-melanocyte-stimulating hormone and substantial inhibition of the synthesis process of melanin, tyrosinase-related protein 1, tyrosinase-related protein 2, and tyrosinase in the body. Through intracellular reactive oxygen species and cell viability studies, THC also prevented oxidative stress caused by H_2_O_2_ in a human keratinocyte *in vitro* cell model. THC was further encapsulated in nanoemulsions comprised of lecithin and examined by using the Franz diffusion cell membrane. Findings suggested that the THC-loaded nanoemulsion formulation permeates more easily across the membrane than a plain THC suspension. Topical administration of THC using lecithin-comprised nanoemulsion efficiently treated melanoma with better penetration efficacy.^113^

### Psoriasis

Psoriasis is a long-term inflammatory multifactorial skin disorder condition driven by hyperproliferative epidermis responses due to the development and hyperactivation of immature keratinocytes.^114−117^ The recent investigation on patients with psoriasis includes ethosomal and liposomal formulations loaded with anthralin to improve its efficacy and safety. These formulations have been integrated into different gel bases to ease their administration. *Ex vivo* permeability tests found that in comparison with the liposomal gel anthralin ethosomal gel had substantially greater permeation via rat abdominal tissue. The clinical evaluation results of these formulations demonstrated minimized side effects of the drug, and anthralin-loaded ethosomal gel in psoriatic patients proved to be an efficient treatment.^118^ For psoriasis treatment, Rapalli et al. formulated the apremilast (API) loaded SLNs further incorporated in topical hydrogel using hot emulsification and size reduction techniques with reduced undesired effects of encapsulated drugs. The prepared formulation was examined *ex vivo* using goat ear skin and HaCaT cell lines. SLN formulation provided improved skin permeation and retention efficiency, extended release of encapsulated drugs, and enhanced therapeutic action against psoriasis by minimizing levels of TNF-α miRNA along with reduced systemic absorption, which could be employed for future clinical manifestation.^119^ Hydrogels composed of clobetasol propionate (CP) embedded nanosponges were synthesized to prevent the undesired effects associated with CP, for instance, steroidal acne, ACD, skin atrophy, and hypopigmentation. The prepared formulation possessed the ability to control the release rate of encapsulated drug and antioxidant activity in THP1 (human leukemia monocytes) cell lines and excellently control the proliferation of keratinocytes and parakeratosis without any adverse effects in the tail of adult mice. Hence, it can be used topically to treat psoriasis.^120^ Liposomes containing metformin were prepared by Jenabikordi et al. by thin-film hydration technology to treat psoriasis by a topical route. Ginger extracts were added to the optimized liposome formulation and evaluated *ex vivo* and *in vivo* in a psoriasis-induced skin model. Liposomes containing metformin increased permeation efficiency and provided localized delivery of the enclosed drug. The synergistic action of ginger and metformin healed psoriatic lesions more rapidly as compared to betamethasone ointment by reducing the levels of TNF-α and IL-6 inflammatory cytokines.^121^ Topically applied Carbopol-based liposomal gel, coencapsulated with curcumin and ibrutinib, was prepared by Jain and his team for the synergistic approach to enhance the efficacy against psoriasis. The prepared liposomes depicted nontoxicity and provided synergistic action and controlled the drug release pattern with better entrapment efficiency. Liposomal gel formulation lowered the Imiquimod-induced hyperplasia of the epidermal layer, psoriatic area, severity index, psoriatic lesions, and ear thickness in the rat model, along with the inhibitory release of cytokines causing inflammation (TNF-α, IL-22, and IL-17) compared to the free form of drug present in a conventionally applied individual topical gel of both the drugs.^122^

### Vitiligo

Depigmentation of skin accompanied by macules of white color without melanocytes is called vitiligo.^123,124^ It can severely impact a person psychologically and can even cause suicidal thoughts.^125^ The topical formulation of an ethosome-based hydrogel loaded with methoxsalen was fabricated to treat this particular condition. Accumulating ethosomal formulation in dermal and epidermal layers resulted in increased skin permeation. Thus, the formulation improved percutaneous penetration of methoxsalen; therefore, it can be employed for vitiligo treatment.^126^ Elhalmoushy and co-workers synthesized hyalurosomes encapsulated with berberine (BRB) for topical application to improve the permeability in targeted therapy of vitiligo treatment. These nanovesicles were immobilized with phospholipids to enhance their physicochemical profile. *Ex vivo* and *in vivo* studies were performed using human skin and mouse models induced with vitiligo. The prepared vesicular formulation was found to possess a significantly improved concentration of biochemical markers compared to plain BRB, showing effectively improved biological efficacy for clinical employment against vitiligo.^127^ Mahmoud et al. designed oleyl alcohol comprising transethosomes strengthened with 8-methoxy psoralen (8-MOP) for photodynamic therapy (to address the adverse effects and solubility limitations of the oral administration of 8-MOP for vitiligo treatment). The transethosomes exhibited high drug loading, entrapment efficiency, deformability index, and *ex vivo* skin permeation ability. When applied topically, it improved NB UVB rays’ efficacy in vitiligo therapy with a nontoxicity profile.^128^

## Nanocarriers for Topical Drug Delivery

**Figure 3:**
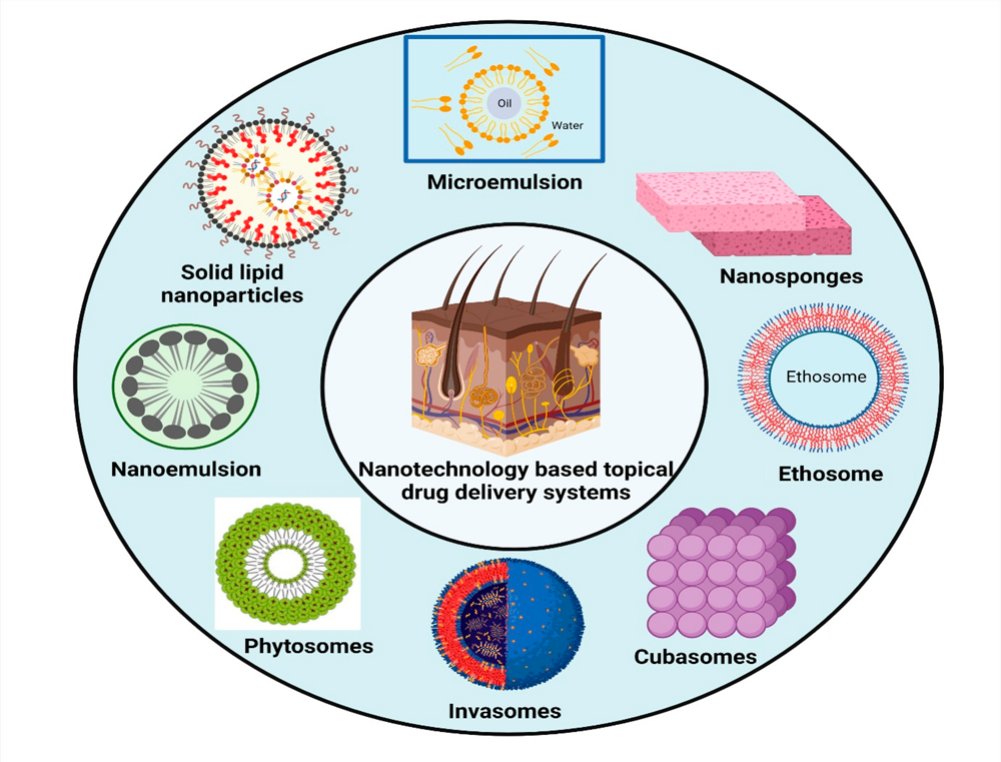
Various nanocarriers employed in topical drug delivery.

Nanocarriers are structures having a particle size below 1–100 nm because of their size scale. New properties arise that ultimately help with better therapeutic action. Currently, the focus is on topically applied nanocarriers (Figure 3) as the skin offers a large area for applying such systems. For dermal treatment, it is essential to distinguish between the desired effects of the formulation: the local effect on the top or inside of the skin (penetration only) or the systemic effect followed by skin permeation.^129^ It is also possible to differentiate between the goals of the production of nanocarriers, which could be the safety of the active ingredients, the targeted delivery to the desired organ, and the controlled release of the active ingredient. As the most promising revolutionary research field of drug delivery,^130^ the topical route is now competing with oral therapy, with about 40% of drug delivery candidate products under clinical evaluation linked to dermal systems.^131^ Due to their enhanced stratum corneum penetration and targeting capabilities, the nanocarriers especially have drawn interest. Skin functions as a negatively charged membrane, so the existence of charge on the nanocarrier surface affects drug diffusion via the skin.^132^ In addition to improving skin permeability and improving skin distribution to certain skin organelles, nanocarriers provide the necessary potential for topical delivery.^1,2^ The subsequent section mentions the widely used nanocarriers (Table 2) for topical therapy.

**Table 2:** Nanocarriers for Topical Drug Delivery

Drug	Nanoformulation	Preparation technique	Size	Inference	Reference
Genistein	Microemulsion	Microemulsification	>100 nm	Drug-loaded topically applied microemulsion improved the bioavailability and therapeutic efficacy of genistein for skin whitening	(133)
Chlorhexidine digluconate (CHG)	Nanoemulsion-based *in situ* dressing	High speed homogenization and ultraprobe sonification	257.5 ± 12.4 nm	Improved penetration and controlled delivery of CHG for treatment for skin infections	(134)
Magnesium ascorbyl phosphate (MAP)	Ethosomal gel	Cold method	160.57 ± 13.7 nm	Controlled permeation and higher retention within skin layers with an excellent decrease in skin melanin content	(135)
Curcumin (CUR)	Permeation enhancer nanovesicles (PE-NVs)	Modified ethanol injection	-	Effectively reduced the skin lesions in hyperpigmented skin of rabbit	(136)
Dexamethasone (DMS)	Cubosomal gel	Emulsification	250.40 nm	Prolonged and sustained drug delivery via topical administration for vitiligo therapy	(137)
Solasonine, Solamargine	Liposomes	Thin-film hydration	220 nm	Improved bioavailability and therapeutic efficacy *ex vivo* using SCC-25 and HaCaT cell lines for skin diseases	(138)
Butenafine (BN)	Bilosome-based topical hydrogel	Thin-film hydration	215 ± 6.5 nm	Considerable effectiveness against skin infections caused by *A. niger* and *C. albicans* fungal strains	(139)
Azelaic acid	Cyclodextrin nanosponges	Melt method	-	Improved activity against oxidation, microbial skin infections in the HaCaT cell lines with safe and sustained release in the treatment of acne and skin pigmentation	(140)
Acyclovir	Lipid-coated chitosan nanocomplexes	Self-assembly method	177.50 ± 1.41 nm	Improved skin permeation and deposition efficiency and significant efficacy in the treatment of skin viral infections	(141)

### Microemulsions

Microemulsions are colloidal dispersions, thermodynamically stable, and shaped without any energy input. Microemulsions emerge, mixing a suitable amount of a surfactant framework and a lipophilic and a hydrophilic component.^142^ They can occur over a broad range or only in narrow ranges depending on the components involved in the device.^143^ The process of formation includes a highly fluid interfacial film and low interfacial tension between the oil and the aqueous phase. Recently designed microemulsions for acute skin inflammation, having vitamin E and vitamin A, demonstrated stability after 30 days, no cytotoxicity in cells, and decreased TNF-α levels as well as inflammation scoring in the dermis. So, the results suggest the potential of microemulsions containing vitamin E and vitamin A for inflammatory skin disorders.^144^ Sharma et al. fabricated microemulsions embedded with 5-fluorouracil (5-FU) for topical administration to treat skin cancers of different types and prevent side effects of 5-FU. This microemulsion is further converted into a gel form and applied on human cadavers and goat skin for *ex vivo* evaluation. The prepared microemulsion gel formulation exhibited enhanced permeation, increased flux, and drug infusion into goat and human cadaver skin with lower irritation than that for free 5-FU. Hence, this microemulsion could be employed as a potential drug carrier system in various forms of skin cancer for future applications.^145^ El-Gogary et al. encapsulated oleuropein within a microemulsion formulation to increase oleuropein’s permeation efficiency for treating skin disorders like psoriasis. Oleuropein-loaded microemulsion showed improved permeation and drug deposition into deeper layers of the psoriatic skin of patients. The prepared formulation showed more excellent activity against psoriasis as compared to the market product (Dermovate cream) of clobetasol propionate during clinical studies carried out on psoriatic patients. The microemulsion formulation reduced the psoriasis area and severity index and improved the clinical therapy index in psoriasis treatment.^146^ Biocompatible fluconazole-entrapped microemulsions comprised of phytomedicinal agents’ oregano, clove oil, or cinnamon were prepared to improve the permeation and therapeutic activity of loaded antifungal drugs across the skin. This microemulsion also contributed to self-antifungal action, and chitosan was added to convert the microemulsion into gel form (MEGELs) to improve skin adhesion. The prepared MEGELs with intrinsic antifungal activity exhibited higher drug release with an increased rate of inhibition of the fungal zone and demonstrated an effective carrier system for fluconazole delivery via topical administration.^147^ Gandhi et al. prepared microemulsions embedded with clove oil using a phase titration technique for therapeutic action against superficial skin fungal infections. The prepared microemulsion-based gel exhibited nonirritation and drug retention at the site of fungal infection with equal activity against fungal infection to that of closet gel during *ex vivo* and skin irritation studies carried out on the Wistar rat skin *in vivo* model. Clove-oil-loaded microemulsion-based gel was found to be significantly effective, stable, and safe and hence could be used as a promising drug carrier in fungal and other skin infections.^148^ From the above studies, it can be concluded that microemulsions possessed lots of advantages like less toxicity, prevented systemic toxicity, and high penetration ability in the skin delivery of drug at a controlled release rate. However, researchers still need to focus on the patentability and commercialization of microemulsions as topical nanocarriers in treating skin diseases.

### Nanoemulsions

They usually consist of an oil system dispersed in an aqueous system, disseminated in an oil network but with nanometer-sized particles or even other greasy stages. Nanoemulsions are homogeneous scattered solutions of two separate liquids, which strengthens the drug’s functionality, such as solubility, durability, permeability, and bioavailability, by encapsulating it into droplet core oil and water. Nanoemulsion has been effective in supplying the active drug at therapeutically relevant levels to the target organ, with minimal pain and adverse effects, and effective in supplying the active drug at therapeutically relevant levels to the target organ. Nanoemulsions have more vital propagating properties in the skin than conventional emulsions, which is why they are used in dermatology to enhance the distribution of medications to and from the stratum corneum. Nanoemulsions have been well used for numerous drugs, including pharmaceuticals, phytopharmaceuticals, cosmeceuticals, and nutritional and nutraceuticals, to improve human health.^149−151^ A study was done on the topical drug delivery of the carcinogenic agent piperine (piperlongumine) utilizing nanoemulsions (NEs) made from alginate and chitosan. The formulation improved drug absorption, and piplartine’s impacts on three-dimensional cancer cells were concentration-dependent; at 1%, it destroyed the epidermal. These results support the potential of piplartin-containing chitosan-modified nanoemulsion as a novel strategy for the topical treatment of acute and chronic inflammatory skin cancer.^152^ Kawakami et al. fabricated a topical nanoemulsion (NE) loaded with oleoresins (SO) using the low energy method with the aim of improving permeation through the skin for the treatment of *Cutaneous leishmaniasis* (CL). *In vivo* studies were performed on a BALB/c mice model induced *with* Leishmania amazonensis. The prepared SO-NE exhibited enhanced penetration and diffusion ability across the skin, reduction in lesions by elimination of inflammation, and amastigotes from protozoan as compared to plain SO solution. SO-NE topical application with simultaneous intraperitoneal administration of meglumine antimoniate provided synergistic action against CL. Findings proved this study as an excellent approach for treating CL via the topical route of administration.^153^ Nanoemulsion (NE) loaded with the antifungal drug miconazole (M) was fabricated using the method of spontaneous titration by Farooq and co-workers to enhance miconazole’s permeation through the skin on topical administration. The prepared M-NE exhibited good stability and excellent dermal permeation, significant therapeutic activity against cutaneous fungal infections caused by *C. albicans* during *in vivo* and *ex vivo* examinations done on female Swiss albino mice animal models as compared to commercially available miconazole formulation. Hence, nanoemulsion could be employed for the topical delivery of antifungal agents for future clinical applications.^154^ A topical nanoemulsion comprising a mixture of virgin nutmeg and coconut oil, loaded with cyclosporine, was designed to treat psoriasis. The prepared nanoemulsion showed improved biological activity, low systemic toxicity, inhibited colony formation, and skin hydration to the HaCaT primary keratinocytes. Therefore, this can be an established topical nanoemulsion as a potential candidate for the treatment of psoriasis and improve the skin’s condition.^155^ Nanoemulsions enclosed with tacrolimus were prepared using the process of spontaneous emulsification and a high-pressure homogenization method in combination, optimized and characterized for the treatment efficacy of psoriasis. The optimized nanoemulsion was further incorporated in a 1% solution of Carbopol to make NE-based gel. The finally prepared gel formulation provided greater skin permeation and retention capability when applied topically on the *in vivo* mice model and provided considerable enhancement in activity against psoriasis as compared to simple market tacrolimus ointment. These NE-based gels also reduced the concentration of inflammatory cytokines in the skin (IL-6 and TNF-α), providing anti-inflammatory action. Hence, this formulation could be employed for the effective treatment of psoriasis.^156^ Thus, these systems are considered to provide better skin penetration, reduce cytotoxicity, improve biocompatibility, control drug size and drug solubility, and improve stability.

### Solid Lipid Nanoparticles

Solid lipid nanoparticles (SLNs) are emulsion-based carriers of lipophilic bioactive compounds, have biocompatibility, and are considered as efficient carriers for antimicrobial agents.^157,158^ Solid lipid nanoparticles entrapped with itraconazole (ITZ) were developed by Kumar et al. using a high shear homogenization method to cure fungal skin infections. The optimized drug-loaded SLNs were further converted into hydrogel formulations that exhibited better gelling rheology and spreading ability. The prepared SLN-based hydrogel provided more efficacy against fungal skin infections and no skin irritation than traditionally used topical and oral antifungal products in the rodent skin model. Findings revealed that this ITZ-SLN-hydrogel formulation possessed great activity against topical skin infections and can be employed for future clinical applications.^159^ Solid lipid nanoparticles enclosed with ketoconazole (KTZ) were designed and optimized by Ramzan et al. and targeted to enhance skin permeation for skin infections. The optimized formulation was photostable and possessed the ability to permeate more profoundly into the stratum corneum of humans. These SLNs also provided drug delivery at a slow and sustained rate and higher skin permeation efficiency compared with the marketed formulation of 2% KTZ during *ex vivo* and *in vivo* studies carried out on rat skin.^160^ Singh and his team prepared miconazole (MCN) loaded SLNs for topical administration and treatment of cutaneous fungal infections. The drug-loaded SLNs were further mixed with Carbopol to make a hydrogel formulation. The resultant formulation exhibited increased MCN retention in rat skin, localized controlled drug release at the fungal site, significant antifungal activity against *Candida albicans*, and excellent antifungal activity during *ex vivo*, *in vivo*, and no-irritation skin irritation studies using rat skin in comparison to the marketed formulation. Hence, the proposed study illustrated that H-SLNs comprised potential future MCN delivery applications via the topical drug administration route.^161^ Kazim et al. formed an ebastine-loaded SLN-based topical hydrogel formulation using chitosan as a gelling agent for the treatment of allergic contact dermatitis (ACD). The prepared hydrogel formulation delivered the drug in a sustained manner, improved penetration efficiency, and increased therapeutic activity against the ACD-induced BALB/c mice model. Conclusively, the developed formulation was found to alleviate the symptoms of ACD.^162^ However, clinical application remains a challenge, which comes up against issues such as the complexity of regulatory requirements. In addition, there is still little *in vivo* knowledge about the ability of these nanosystems to permeate biological membranes, distribute the drug in the skin strata, and deposit themselves in the body’s tissues.

### Microsponges

Microsponges are considered an advanced system for the delivery of a drug capable of encapsulating various agents like antiacne agents, essential oils, anti-inflammatory agents, and fragrances.^163,164^ Their diameter ranges from 5 to 300 μm, and microcarriers are chosen as a medium for the topical delivery of medicines as they can prolong drug duration.^165^ Microsponges are efficient carriers capable of decreasing the unwanted local effects of dermatological agents. Naringenin (NG)-loaded microsponge gel using polymer ethyl cellulose is produced for dermatitis. Carbopol was added to the optimized microsponge batch and was incorporated into the 1% naringenin-loaded microsponge gel (NGMSG1%). *In vivo* studies were conducted on albino Wistar rats. In animals, no skin irritation was observed with NGMSG, and NGMSG 1% gel demonstrated healing at a faster rate, a considerable decrease in swollen earflap thickness, WBC count, and elevated drug deposition. Therefore, this microsponge gel can be used for atopic dermatitis treatment.^166^ Jakhar et al. fabricated ethylcellulose microsponges of dapsone using the quasi-emulsion solvent diffusion method, further incorporated into Carbopol to make a topical gel formulation. Bacterial strains like *Staphylococcus aureus* and *Propionibacterium acnes* that cause acne were used for evaluation of therapeutic activity against acne. The results depicted that the developed microsponge topical gel formulation was in the micrometer size range; had a uniform, spherical, and spongy texture; released more than half of the total concentration of the entrapped drug; had better efficiency for drug encapsulation; and exhibited significant activity against the above bacterial strains. Hence, it could be employed for the treatment of acne via topical application.^167^ Sultan et al. loaded the antifungal drug Luliconazole (LCZ) into microsponges of ethyl cellulose and Eudragit RS 100 (drug–polymer ration) by the quasi-emulsion solvent diffusion process, further incorporated in Carbopol gel to provide the drug release at a controlled rate. *In vitro* evaluation of microsponges suggested that an increased concentration of polymer increased the drug entrapment efficiency. Microsponge-based Carbopol gel provided controlled drug delivery and possessed excellent potential against fungal infections of derma, for instance, tinea cruris, tinea corporis, and tinea pedis.^168^ Thus, microsponges have the unique advantage of controlled release and are biologically harmless. This technology allows for the trapping of chemicals, which is thought to lessen the adverse effects and improve the stability, elegance, and formulation flexibility.

### Nanosponges

Nanosponges are hyper-cross-linked cyclodextrin polymers that are nanostructured to form 3D networks and are acquired with a cross-linker-like carbonyldiimidazole by complexing cyclodextrin. In their crystal architecture, numerous polymer chains can construct unique microdomains appropriate for coencapsulating two drugs of distinct chemical compositions.^169^ Nanosponges dominated the lead because they effectively solubilize poorly water-soluble drugs and have extended release simultaneously, and because of their inner hydrophobic cavities and external hydrophilic branching, they can load both hydrophilic and hydrophobic drug molecules. These tiny sponges will circulate within the body to accomplish precisely targeted delivery before they encounter their target location to activate the drug in a controlled and configured manner. There are many implementations of nanosponges in topical drug delivery to enhance the efficacy, durability, permeation, and bioavailability of drugs.^170^ Econazole-enclosed nanosponges are comprised of *N*,*N*-carbonyldiimidazole and β-cyclodextrin as a cross-linking agent, and the organic polymer was fabricated using the melting technique by Srivastava and co-workers for the treatment of fungal skin infections. The optimized nanosponges were further incorporated in the hydrogel (EN-TG) of Carbopol 934, and the permeation enhancer used was pyrrolidone. The prepared nanosponge-based hydrogel permeated more rapidly across the goat skin and had greater efficiency in preventing the growth of a fungal infection as compared to the market product during *in vitro* evaluation. It also depicted excellent activity as an antifungal agent during *in vivo* studies conducted on male Albino Wistar rats.^171^ Harsha et al. designed the antifungal drug sertaconazole containing nanosponges for topical application. The nanosponges were prepared using the emulsion solvent diffusion technique, including polymers like poly(methyl methacrylate) and ethyl cellulose, poly(vinyl alcohol) as the surfactant, and dichloromethane as the cross-linking agent. The optimized formulation displayed targeted and controlled release of drug for a longer period, minimized the adverse effects of formulation, and reduced dosing frequency and drug retention in the layers of skin during *in vitro* evaluation. Hence, this nanoformulation could be employed topically for several skin problems like dermatophytosis, candidiasis, athlete’s foot injuries, etc.^172^ Ahmed et al. designed four different types of nanosponges to encapsulate the antifungal drug butenafine. The designed nanosponges contained poly(vinyl alcohol) and ethyl cellulose, respectively, and were prepared through the solvent emulsification method. These nanosponges were mixed with 1% Carbopol gel to convert them into final topical hydrogel formulations. The prepared nanosponges containing hydrogel provided deep penetration into the dermal layers, targeted and controlled drug release, and increased antifungal activity with extended dosing intervals compared to commercially available fungicidal cream against the strains of *A. niger in vitro*. Therefore, these nanosponge-based hydrogels can be used for targeted drug delivery via a topical route with minimum skin irritation, improved antifungal efficiency, and prevented side effects.^173^ Hydrogel comprised of bifonazole-encapsulated nanosponges was formulated by Krishna et al. to treat fungal infections. Emulsion solvent diffusion methodology was used for the formation of nanosponges, and optimized nanosponges were incorporated into a gel for topical application and controlled the release rate of the loaded drug. The nanosponge-based hydrogel formulation exhibited drug release at a sustained rate, nontoxicity, and improved antifungal activity and was a nonirritant to the rat skin model. Based on the findings of the current work, it could be established that this formulation could be employed for future application in fungal problems treatment.^174^

## Vesicular Drug Delivery Systems

Much scientific work has been conducted to develop and investigate vesicular drug carrier structures in the past two decades. These are of great interest, particularly colloidal lipid aggregates. However, the mode of action of lipid vesicles in topical drug delivery gives some promising findings indicating that vesicular nanosystems remain suitable carriers for topical drug delivery.^40^

### Liposomes

Liposomes are lipid bilayer structures that can carry therapeutic agents of hydrophilic nature between the core bilayer and lipophilic drugs. Numerous liposome-based medications and biomedical products have been approved for medicinal use in recent years since they are nontoxic.^175^ Melatonin (MLT) embedded elastic liposomes employing the method of thin-film dispersion were formulated to slow down the process of skin aging. These liposomes were prepared with the aim of enhancing the poor solubility and bioavailability limitations associated with the oral administration of MLT. The prepared elastic liposomes crossed the UV-induced photoaged skin layers of the female Kunming mice model more efficiently than liposomes utilized conventionally, improving dermal hydration and elasticity by maintaining collagen and fibers of derma during *in vivo* evaluation. Hence, it could be employed successfully for the future application for encapsulation of poorly soluble and low bioavailable therapeutic agents for treating skin diseases.^176^ Since the term “liposome” has a broad definition that encompasses several types of vesicles with typical sizes up to several micrometers, nanoliposomes have been referred to as nanoscale bilayer lipid vesicles.^177^ Clove essential oil (CEO) and tea tree oil (TTO) were combined in varied concentrations and put into multicompositional nanoliposomes. It was expected that EOs in bulk and nanoliposomes containing multifunctional components loaded with EOs would be effective. Trichophyton rubrum fungus was used as a test model to determine the antifungal effectiveness of manufactured EO-loaded nanoliposomes. The CEO-loaded nanoliposomes displayed a maximum entrapment efficacy of 91.57 2.5% in comparison to TTO-loaded nanoliposomes. The CEO-loaded nanoliposome fraction demonstrated the highest MGI when tested against *T. rubrum* strains.^178^

### Niosomes

Niosomes are nonionic surfactants, a new vesicular delivery mechanism that is thermodynamically stable and can be used to deliver drugs on a continuous, controlled, and targeted basis. Niosome vesicles are formed only when surfactants and cholesterol are correctly combined and can be broken into unilamellar, oligolamellar, or multilamellar structures.^179^ Niosomes are microscopic lamellar structures, ranging in size from 10 to 1000 nm, which are again similar but more compact than liposomes, with this durability being the product of their bilayer formation by nonionic surfactants. Niosomes can be delivered via nearly all transmission channels, such as nasal, parenteral, transdermal, ocular, and pulmonary, for example, due to their high biocompatibility and improved distribution to individual tissues.^180^ Pentoxifylline (PTX) is reported to accelerate the repair; thus, the liposomal formulation of PTX was fabricated for wound repair and finally incorporated into the cream. Penetration of PTX was increased in liposomal formulations as compared to its conventional cream. An animal study in BALB/c mice demonstrated that PTX-niosomal creams helped in minimizing the healing duration. The final wound size was also reduced, which defines the potential liposomal formulations.^181^ The topical gel of Carbopol, comprising cyclosporin-encapsulated niosomes (using the method of film hydration), was prepared by Pandey et al. for psoriasis treatment. Male albino rats’ abdominal skin was used for *ex vivo* studies for niosomes encapsulated with cyclosporin, Carbopol liposomal gel, and cyclosporin suspension, which resulted in a higher permeation rate and increased stratum corneum deposition of niosomes than cyclosporin suspension. *In vivo* studies carried out using male Swiss albino mice of the Imiquimod-induced psoriatic plaque model demonstrated a greater reduction in severity index and psoriatic area by niosomal gel as compared to the plain cyclosporin suspension. So, these niosomes could be employed for potential psoriasis therapy.^182^ Ultrasonicated niosomes loaded with arbutin were formulated by Radmard and co-workers to enhance drug delivery through a topical route in treating skin hyperpigmentation. The prepared drug-containing niosomes were free from *in vitro* cytotoxicity, permeated rapidly, and deposited more in the skin of male Wistar rats than simple arbutin-containing topical gel. They gave an increased viability of cells in the HFF cell lines and were also nonirritating to the male Wistar rats during an *in vivo* examination. Hence, they could be employed for better therapeutic efficacy against skin pigmentation with minimal systemic toxicity and skin irritation.^183^ Ceramide-based topically applied niosomes, also called centrosomes coencapsulated with nicotinamide (NIC) and methotrexate (MTX), were prepared through the ethanol injection method for efficacy enhancement and toxicity reduction in psoriasis therapy. The prepared drugs containing the formulation exhibited enhanced permeation and drug deposition efficacy in Sprague–Dawley rats’ abdominal skin and apoptosis by stopping the cell proliferation at the S-phase in human keratinocyte cell lines. The topical application of centrosomes on Sprague–Dawley rats (induced with imiquimod psoriasis *in vivo* model) ameliorated psoriatic lesions, reduced the thickness of the epidermis and the spleen index, and minimized proinflammatory cytokine (IL-6, IL-22, IL-23, IL-17A, IL-1β, TNF-α) effects on mRNA as compared to orally administered MTX solution without any systemic toxicity. This codelivery approach with centrosomes could be an excellent carrier system for topical application, rich in advantages for treating psoriasis.^184^

### Ethosomes

The delivery of medications via a topical route, commonly referred to as skin drug delivery, is an attractive solution to the treatment of many diseases. An adequate dosage in the skin tissue is typically achieved by providing high doses, which could potentiate the side effects of the drug. As a lipid vehicle, ethosomes have demonstrated excellent efficiency in the delivery of percutaneous medicines. Ethosomes are extremely deformable ethanolic rounded vesicles, which may maximize the volume of substance that penetrates the stratum corneum and deposits deeper skin layers. Ethosomes are an excellent improvement in permeation and selective drug release.^185^ Ethosomes can deliver both agents in more significant concentrations through the stratum corneum and deposit the compounds within the skin layers. Ethanolic ethosomes surround vesicles consisting of phospholipids, ethanol, and propylene glycol. They have specific characteristics that render them a very desirable instrument for enhancing drug penetration through the skin, such as smaller particle size, strong deformability, strong stability, and low toxicity.^186^ Srivastava et al. prepared ethosome nanovesicles for the delivery of enclosed berberine chloride dihydrate (BCD); optimized ethosomes were added to Carbopol solution to prepare topical gel as a final formulation for the treatment of dermatitis. The developed ethosomal gel penetrated more deeply across dermal layers of rats and inhibited zones of *Pseudomonas aeruginosa*, *Staphylococcus aureus*, and *E. coli*. These ethosomes were also free from skin irritation and significantly lowered the concentration of the cell nucleus that caused skin inflammation. So, findings revealed that BCD-containing ethosomes, when applied topically, possessed excellent efficacy for dermatitis therapy.^187^ Ethosomes enclosing lomefloxacin were prepared by a cold method and evaluated by El-Hashemy. The optimized formulation was further incorporated into Carbopol solution, forming a gel form for topical application to treat skin infections. The prepared ethosomal gel provided better penetration, skin accumulation, and controlled release of loaded drug as compared to the solution containing free drug during *in vitro* and *ex vivo* examinations. Hence, these ethosomes provided more incredible therapeutic action against skin infections and could be employed in the future for the treatment of skin disorders by the topical route.^188^ Ismail et al. designed an HPMC-based topical gel comprised of brucine-enclosed ethosomes for the treatment of skin cancer by thin-film hydration techniques. The prepared ethosomal gel provided a more sustained release of an encapsulated drug than ethosomes and was nontoxic to A375 skin cancer cell lines during *in vitro* evaluation and excellent *ex vivo* dermal penetration across male Wistar rat skin than brucine suspension. The findings suggested these ethosomes as a potential alternative to the topical gel used conventionally.^189^ Guo et al. conjugated the glycyrrhetinic acid and d-α-tocopherol acid polyethylene glycol succinate, grafted them on the surface of curcumin-encapsulated ethosomes, and formed multifunctional ethosomes for the treatment of psoriatic skin. *In vivo* evaluation for therapeutic efficacy against psoriasis was performed on imiquimod-induced BALB/c psoriatic mice. The findings provided significant damage suppression due to the oxidative stress and expression of inflammatory cytokines, increased skin permeation ability, cellular uptake, skin deposition, sustained release of the drug, and overall enhanced therapeutic efficacy for the treatment of psoriatic skin.^190^

### Transfersomes

Transferosomes are ultradeformable vesicles composed of a lipid bilayer with phospholipids, an edge activator, and an ethanol/aqueous core, and the deformity of liposomes can be accomplished by using surfactants in sufficient ratios to increase the skin permeation of drug molecules. Ethanol has significant factors (up to 10%), lipids (5–10%, soy phosphatidylcholine or egg phosphatidylcholine), and surfactants (10–25%, edge activator (EA)) and an aqueous compartment enclosed by a lipid bilayer and buffering agent (pH 6,5–7) (as a hydrating medium, e.g., saline phosphate buffer pH 6.4).^191^ Transferosomes are able to enter entire deeper skin regions following topical administration compared to liposomes, providing more significant amounts of active substances, rendering them an effective topical drug delivery carrier. Phosphatidylcholine is the most abundant lipid portion of the cell membrane in most transferosomes and is thus highly tolerated for the skin, minimizing the possibility of adverse results, such as hypersensitive skin reactions. Transferosomes are widely investigated among these vesicular carriers and are recently becoming necessary because of their ability to resolve permeation difficulties via the stratum corneum.^191,192^ Topical garlic oil (GO) containing finasteride (FI) encapsulated nanotransferosomes (NTFS) was prepared by Hosny and his team to avoid the adverse effects of FI on oral administration. The optimized NTF formulation was added in aloe vera gel for the action of aloe vera and GO against the *Propionibacterium acnes* prevention of microbial growth at the scalp during the treatment of alopecia areata. The FI-GO-NTF-based aloe vera gel provided sustained and targeted drug delivery with increased encapsulation efficiency, nontoxicity, nonirritatant, and significant inhibition of the microbial zone of the said bacterial colony and more effectively treated the alopecia areata. Conclusively, these preparations can control the bacterial attacks during the alopecia areata therapy and make the treatment effective and safe so that it can be employed successfully for future endeavors.^193^ Topical Carbopol hydrogel loaded with optimized triamcinolone acetonide encapsulated transferosomes (TA-TFS) using the method of thin-film hydration was prepared by Yadav and co-workers for the treatment of psoriatic skin. TA-TFS loaded topical gel sustained the release of the drug for a prolonged period compared to TA-TFS suspension and provided better penetration and drug retention *ex vivo* carried out on BALB/c mouse dorsal skin. Also, the prepared gel possessed the ability to more effectively treat the psoriatic lesions and lower the severity index and swelling of psoriatic skin by lowering the expression of inflammatory cytokines, for instance, IL-17, IL-23, and IL-10, in an *in vivo* imiquimod-induced Wistar rat skin psoriasis model. Thereby, these formulations could be employed as a choice of conventional topical formulations for the effective treatment of psoriasis with controlled, targeted, and nonirritation advantages.^194^

### Invasomes

Invasomes are modern elastic phospholipid vesicles that display improved percutaneous penetration compared to traditional liposomes. In their shapes, these vesicles include phospholipids, ethanol, and terpene. The mixture of all these ingredients symbiotically enhances the medication quantity in deeper layers of the skin and the mobility properties of soft vesicles. The potential to penetrate across skin layers increases intrusive behavior by liquefying the bilayer structure of SC lipids and disrupting interactions between lipids and intracellular proteins. Invasomes with both hydrophilic and lipophilic products have been found to be effective drug delivery mechanisms.^195^ Itraconazole-loaded, valencene-containing invasomes were prepared and further incorporated in 1% Carbopol gel for its topical translingual and therapeutic application against nail skin fungal infections. The optimized formulation was tested and compared with Azrith 1% gel (marketed product) for antifungal and skin permeation efficacy using strains of *Trichophyton rubrum* (common onychomycosis causative pathogen) and goat hooves, respectively. The prepared formulation of itraconazole provided better skin permeation efficiency and therapeutic efficacy against nail fungal infection than the conventionally used marketed product. Hence, it could be employed as a choice in place of conventional products.^196^ The present work is comprised of designing the topical invasomes enclosed with vismodegib (VSD) to avoid the side effects of VSD on oral administration and further incorporated in Carbopol gel for bioavailability enhancement during the treatment of skin cancer. The invasome formulation was also composed of ethanol and terpenes for their synergistic action to enhance skin permeation and compared with the liposomal formulation. The prepared formulation was free from toxicity and skin irritation and provided sustained VSD release during an *in vitro* examination. Also, the VSD-encapsulated invasome gel exhibited enhanced permeation and drug deposition across the dermal layers of adult male rats *in vivo* studies. So, the invasome formulation possessed the potential of future application for topical application with improved therapeutic efficacy with minimal side effects.^197^

### Cubosomes

Cubosomes are nanocomposite liquid crystalline structures that are made up of amphiphilic lipids, regarded as biocompatible drug delivery carriers. Due to the similarities between the inner configuration and the epithelium cell, cubosomes can enter the skin and mucosa, increasing drug bioavailability.^198^ Cubosomes have a rare ability to encapsulate and defend protein and peptide drugs from deterioration. They can easily capture hydrophilic, hydrophobic, and amphiphilic compounds due to their high internal surface area per unit volume and a three-dimensional arrangement of hydrophilic and hydrophobic domains.^199^ Cubosomes were prepared for antimicrobial peptide LL-37 delivery by topical route. The antibacterial action of cubosomes was evaluated through an *ex vivo* pig skin wound infection model with *Staphylococcus aureus*. It was revealed through proteolysis studies that LL-37, while associated with the cubosomes, was completely shielded from enzymatic attacks, which also denotes a strong peptide–particle interaction. No skin irritation occurred with cubosomes, hence allowing its administration by the topical route. The results of the *ex vivo* wound infection model depicted that LL-37 in preloaded cubosomes destroyed bacteria most efficiently.^200^ Nanocubosomes were formulated by Salem et al., for enhancement of skin permeation and therapeutic availability in treating wounds of encapsulated rosuvastatin calcium (RSV). For synergistic action, optimized nanocubosomes were further capped in AgNPs; also, the desired quality possessing AgNPs were embedded in a Carbopol gel for topical application. The *in vivo* evaluation was performed on the female Wistar rat animal model. The prepared gel of AgNPs loaded with RSV released drug in a sustained manner compared to gel containing free drug and was free from any type of edema and skin irritation. This formulation also increased the efficacy of interleukin-1β and tumor necrosis factor-alpha; from that, it was confirmed that these gel form AgNPs of RVS enclosed cubosomes possessed better potential to treat the skin wounds as compared to commercially available gentamicin ointment and could also be used for tissue repair.^201^ Rapalli et al. synthesized hydrogel preparation containing cubosomes enclosed with antifungal agent ketoconazole through a hot emulsification methodology. The prepared hydrogel was evaluated *ex vivo* and for antifungal effectiveness using fresh ear skin of goat and MTCC 1403 cell lines of *Aspergillus flavus*, respectively. The prepared formulation was free from skin-irritation problems and provided sustained drug release and enhanced antifungal activity against the used cell line compared to the marketed product. Hence, it could be employed for future application of antifungal treatment.^202^

### Phytosomes

The phytosome, the term “phyto” means herb and “some” means like the cell, is a carrier that has arisen as a potential method to improve the bioavailability of bioactive components (also known as photophospholipid complexes). Phyto-phospholipid compounds are developed under certain circumstances by conjugating active components at given molar ratios with phospholipids.^203^ Because of their more significant penetration potential through the lipoidal biomembrane, phytosomes provide greater bioavailability relative to traditional herbal extracts.^204^ The phyto-phospholipid complex can be used as a possible transporter to enhance the delivery of topical medications due to its interesting biocompatibility and ability to combine with skin lipids. Thus, phytophospholipid complexes may be designed for different therapeutic purposes after sorting and a number of prospective operational extracts or plants. They may also be used for cosmetics or skin diseases to facilitate the rate of bioactive absorption into the skin in a way that maintains skin morphology.^205^ Centella Asiatica (CA) phytosomal formulations was explored for AD. It was revealed from the histological analysis that CA phytosome inhibited inflammatory cell infiltration along with iNOS and COX-2 expression, NF-dB activity, and TNF-a, IL-1β, and IgE release. From *in vivo* studies it was clear that CA phytosome inhibited LPS-induced DNA binding activities of NF-κB and thus is probably a promising AD treatment.^206^ Kumar et al. formed phytosome containing *Pistacia integerrima* hydroalcoholic extract using solvent evaporation technology to treat scabies. The resulting phytosomal hydroalcoholic extracts of *Pistacia integerrima* were more effective against the scabies-causing organism *Sarcoptes scabiei*.^207^

All aforementioned potential nanocarriers revealed site-specific delivery of the therapeutic agent, reduced dose in addition to dosing rate, enhanced therapeutic efficacy, hydrophilic and hydrophobic drug encapsulation, shielding degradation-prone drugs, etc. These are still in the development stage, considering the possible benefits of these devices, as shown in their preclinical trials. There is a long path to cover ahead of their commercialization.

## Other Nanocarriers for Topical Drug Delivery

### Nanofibers

The utilization of electrospun polymeric nanofibers has shown to be an intriguing method for drug delivery systems. The high surface-to-volume ratio of the fibers can enhance a number of processes, including mass transfer processes, drug loading, and cell attachment and proliferation. The field of drug delivery, for the regulated release of active ingredients, is one of the most significant and researched applications of electrospinning. This approach has the advantage of allowing a wide range of poor solubility medications to be placed into the fibers for improved bioavailability or controlled release.^208^ Nanofibers made of chitosan (Ch)/mel (0.001 and 0.003%) were created for the topical treatment of acne vulgaris. The effects of Ch/Mel nanofibers on the management of *Propionibacterium acnes* were investigated using animal models. The results depicted that the Mel loading into Ch/Mel 0.003% was 86.741%. During 72 h, this Mel-releasing process (89.65%) of Ch/Mel 0.003% was gradual. After being placed into the polymer framework, the Mel maintained its hemolytic activity. The drug toxicity investigation showed that normal human dermal fibroblasts were not significantly harmed by Ch/Mel nanofibers (HDF). In *in vitro* and animal experiments, the Ch/Mel 0.003% group inhibited *P. acnes* growth the most, and it also reduced inflammation and redness the most. For the treatment of acne vulgaris, the Ch/Mel 0.003% structure is suggested as an effective topical medication delivery strategy.^209^ Utilizing the electrospinning technique, the fabrication of composite nanofibers from poly(vinyl alcohol), quercetin, and essential oils was carried out for acne. Results demonstrated a skin deposition rate of 28.24% 0.012, a markedly stronger antibacterial activity against *Propionibacterium acnes*, and complete safety on skin fibroblastic cells. Clinical testing on acne patients revealed that the nanofibers reduced inflammatory, total acne lesions, so it clearly suggests it is a viable topical antiacne delivery strategy.^210^ For the development of antiacne face masks, electrospun poly(vinyl alcohol) (PVA) nanofibers containing ZnO nanoparticles were formed. The sol–gel process was used to make nanoparticles of zinc oxide (ZnO), and then PVA/ZnO composite nanofibers (0, 1, 4, and 7 wt % with regard to PVA) were prepared. Results for antibacterial activity revealed a clear zone of *Cutibacterium acnes* on the PVA/ZnO 7% nanofibers measured at 2.25 mm, suggesting the nanofibers’ antiacne properties.^211^ A tazarotene (TZT)-calcipotriol (CPT)-loaded nanofiber and Carbopol-based hydrogel film were developed in the study. The poly(vinyl alcohol)/polyvinylpyrrolidone (PVA/PVP) K-90 polymeric blend used for nanofiber production and hydrogel films were created by combining it with a Carbopol base. At the end of 72 h, the TZT-CPT-PVA/PVP-NF nanofibers showed 95.68% 0.03% drug release, demonstrating a regulated release pattern and fitting best to Higuchi release kinetics. In comparison to TT-PVA/PVP-NF nanofibers, TZT-CPT-PVA/PVP-NF hydrogel film has a higher potential for the treatment of psoriasis.^212^ Three MTX-loaded electrospun nanofibrous patches were fabricated using a combination of polycaprolactone (PCL), eudragit L100, and both. Each formulation’s *in vitro* drug release profile was then discovered. According to release experiments, the Eudragit L100-PCL formulation with MTX loading outperformed other formulations in terms of mechanical behavior and *in vitro* drug release. The generated controlled-release MTX-loaded electrospun patches appear to hold promise for treating psoriatic plaques on the skin for a longer time.^213^

### Microneedles

Dissolving microneedles (DMNs) were fabricated to allow painless and direct cutaneous medication delivery. They are hydrophilic, mainly polymer-based constructions that can penetrate the skin. By momentarily disturbing the skin’s surface layer, the microneedle delivery device uses the diffusion process to transfer the medicine through the skin. On a tiny patch, hundreds of microneedles are organized to help distribute enough medication to have a therapeutic impact.^214,215^ The trilayer dissolving microneedle array (RBTF-TDMNs) was created to maximize the intradermal administration of RBTF for the treatment of melanoma. It has been demonstrated that RBTF-TDMNs are powerful enough to penetrate excised porcine skin while keeping their physicochemical qualities, quickly disintegrate, and deposit RBTF intradermally. Finally, a dermatokinetic study showed that, in comparison to RBTF dispersion and free drug-loaded TDMNs, RBTF-TDMNs exhibited special delivery efficiency and therefore can be used as an adjuvant tool for the topical therapy of melanoma.^216^ A dissolving microneedle (DMN) containing azelaic acid (AZA) was created and assessed, offering acne patients a unique, self-administered, and quick-acting route. The outcomes showed that AZA-DMN had good formulation stability and skin compatibility. Furthermore, there was no discernible variation in the levels of AZA in the rats’ plasma before and after treatment, indicating that the main site of action for AZA-DMN was local skin. Studies demonstrated that the formulation improved the permeability of AZA, and its observable antibacterial activity on *Propionibacterium acnes* and *Staphylococcus epidermidis* made AZA-loaded DMN a promising therapeutic method for acne.^217^ Monomethoxy-poly(ethylene glycol)-polycaprolactone (MPEG-PCL) nanoparticles loaded with 5-fluorouracil (5-Fu) and indocyanine green (5-ICG-MPEG-PCL) reacted with near-infrared light, and the nanoparticles were integrated with a dissolvable microneedle system (HA MN) containing hyaluronic acid to form 5-Fu-ICG-MPEG-PCL loaded microneedles (HA MN) for skin cancers and melanoma tumors. A dissolvable microneedle could deliver 5-Fu-ICG-MPEG-PCL through the skin, and near-infrared light could control the release behavior of the drug in the nanoparticle, making it possible to cure skin cancer in a single dose, improve skin cancer cure rates, and provide a new idea for treating skin cancer in the clinic.^218^ To prepare the arrowheads for two-stage separable microneedles (MNs), lauric acid and polycaprolactone were used as phase-change materials. Doxorubicin and indocyanine green were embedded in the MNs. A dissolvable support base is used to cap the arrowheads. This base is composed of poly(vinyl alcohol) and polyvinyl pyrrolidone (PVA/PVP). Rapid absorption of interstitial fluid causes PVA/PVP support bases to dissolve in skin tissue, leaving arrowheads intact. NIR irradiation results in the photothermal transformation of the ICG, which liberates and penetrates the DOX from the MNs, abating the embedded arrowheads. This formulation could be a promising approach in the treatment of skin cancer.^219^

### Dendrimers

Dendrimers are highly branched polymers with numerous inner cavities, peripheral groups, and structural characteristics. They are essential in nanotechnology, pharmacology, and medicinal chemistry. Dendrimer terminal functional groups can be chemically bonded to other molecules to change the surface characteristics for uses like biomimetic nanodevices.^220^ For the treatment of melanoma, dendrimeric micelles were created to encapsulate paclitaxel and curcumin. PAMAM dendrimers coupled with histidine-arginine dipeptides and cholesterol were used to create dendrimeric micelles (PHRC). Curcumin had a higher encapsulation efficiency than paclitaxel (80.2% and 76.3% versus 30.7% and 38.2% in PHRC6/TPGS and PHRC23/TPGS, respectively). According to the findings, PHRC/TPGS dendrimeric micelles had a great potential for the nanoformulation containing hydrophobic drugs and showed good bioavailability and bioactivity against skin cancer.^221^ Reactive oxygen species (ROS) are formed by PDT (photodynamic therapy) using a photosensitizer, light, and cellular O_2_ to cause oxidative stress and apoptosis. A photosensitizer known as silicon phthalocyanine Pc 4 has shown promise in treating a variety of skin conditions in clinical testing. PDT works as the agent for the treatment of fungi by converting PC 4. About 13% of the amine chain ends in the vehicle’s poly(amidoamine) dendrimers were PEGylated to increase water solubility and prevent nonspecific adsorption. Study results of *C. albicans* showed that the dendrimer carrier had no effect on Pc 4’s efficacy. During photoactivation, encapsulated Pc 4 efficiently produced ROS and destroyed fungal infections. The findings described that PC 4 has the ability to effectively eradicate solvent toxicity and kill drug-resistant *C. albicans*.^222^

## Patents and Brief Topical Market Overview

Patents provide detailed information and are considered as a highly informative source of knowledge. Knowledge gained from patents plays a crucial role in identifying trends in technology growth.^223^ In general, patent analysis is used to assess competition in technological variations at the industrial or national level, to determine technological strengths and to explore the future international market.^224^ Furthermore, it is easy to predict future developments in technology or a specific industry through patent analysis (Table 3).

**Table 3:** Patents in Topical Drug Delivery

Patent no.	Title	Date of publication	Assignee	Reference
US9439859B2	Adjuvant incorporation in immunotherapeutics	2016-09-13	Harvard College Brigham and Womens Hospital Inc. Massachusetts Institute of Technology	(225)
US10695306B2	Systems and methods for treating vitiligo	2020-06-30	Transdermal Biotechnology Inc.	(226)
US11369664B2	Topical composition for improved healing of open wounds	2022-06-28	Eleos Pharmaceuticals Inc.	(227)
US20200214961A1	Botulinum nanoemulsions	2020-07-09	UMass Lowell	(228)
WO2020102494A1	Nanoemulsion compositions having enhanced permeability	2020-05-22	Bluewillow Biologics, Inc.	(229)
WO2020146415A1	Methods of treating cancer	2020-07-16	Foghorn Therapeutics Inc.	(230)
WO2020150633A1	Gene editing to improve joint function	2020-07-23	Orthobio Therapeutics, Inc.	(231)
WO2020172260A1	Topical composition for improved healing of ocular ailments	2020-08-27	Application filed by BUICE Mona E., SAILORS David M., Woody Jonathan, James Louis Wood, GREESON Joshua Z.	(232)

The market for topical products was estimated at US$92.4 billion in 2020 and is anticipated to grow to US$129.9 billion by 2027.^233^ It is clear that topical generic medication items primarily sold as creams, ointments, lotions, sprays, and solutions rule the market. The different semisolid, liquid, and solid topical delivery products combine chemicals in solutions or dispersions that are water, water-miscible, surfactants, oils, skin-penetration-enhancing, colloidal, propellant, solid, and polymer. The individual needs should be taken into account most when developing each of these topical dose forms; the proper targeting and distribution of medications given in a specific topical dose form will be necessary to address individual skin demands, for instance, acne, immunological stimulation, reducing inflammation, infections, pain relief, whitening, immunological stimulation, hair loss, etc.^234^ Additionally, some products might not be the best choices for use on mucous membranes, near the eyes, or on areas of skin that are lacerated or hairy. The physicochemical characteristics of therapeutic agents are the second factor that needs to be considered when selecting a dosage form for the topical route. It is reasonable to assume that the medications soluble in water will be formulated in aqueous topical delivery systems, which include solutions, lotions, and creams, while the medications insoluble in water need to be dissolved in oily ingredients or solubilized in surfactant systems. The accessibility of inexpensive, efficacious, as well as safe topical products is a major barrier to satisfying the two objectives mentioned above.^235−238^

The fact that the cost of generic topical dermatologic medications increased by 10 times more between 2005 and 2016 than the rate of inflation illustrates this additional consumer requirement. As clinical trials or clinical equivalence investigations are considered the gold standard, regulatory approvals of generic topical medications have generally fallen behind commercial demands.^213^ To deliver the necessary sensitive clinical end point evaluation for addressing variations in skin conditions, product use, placebo response, and body site, in addition to genetic, environmental, ethnic, and other biological differences, such investigations typically include higher subject numbers, time commitments, and costs. Consequently, more thorough research should be conducted to fabricate topical nanobased drug delivery for a commercial setting.

## Conclusion

The recent technological advancements in delivery via the topical route have been transformed due to a deeper understanding of the molecular level structure of the stratum corneum and the permeation pathways through the skin. Thus, more development of target-oriented topical therapies and uncomplicated, noninvasive therapeutic strategies is necessary. The main problem with the commercially available formulations for topical medication administration is that they cause systemic toxicity since the drug is absorbed into the body after being applied topically. Nanotechnology was developed as a simple and nonintrusive solution to these shortcomings. Nanotechnology has emerged as a promising approach for enhancing the skin’s barrier function in recent years. This article discussed commonly used nanoparticulate carriers for enhancing medication delivery through the dermis. Nanomedicines applied topically have exciting options for dermal administration, and current advancements appear to have boosted their therapeutic potential. Vesicles with the necessary customized qualities are produced by improving and changing their composition and structure. The above-discussed findings showed a special relationship between nanoparticulate carriers and skin structures to facilitate drug delivery. With advancements in engineering material, fabrication, and chemical analysis, researchers have focused on the creation of novel nanoparticulate carriers with attractive traits for epidermal implementation.

Further research, however, should confirm the advantages and calculate the risk–benefit ratio for various therapeutic drugs loaded in nanocarriers. In order to dissect the true potential and surplus value of a specific carrier device for a specific indication, close interactions between medications, pharmaceutical technology, and chemistry are required, given the broad range of newly emerging nanocarrier technologies. Beyond the many benefits of using nanotechnology in topical drug delivery for skin conditions, there are many issues with the clinical translation and commercialization of these cutting-edge topical formulations. The stability issues of the generated formulations, scale-up constraints, high costs, and reproducibility issues of the synthesized nanoparticles are the key hurdles for the clinical translations of nanomedicine. Additionally, executing many characterization and assessment procedures, clinical efficacy tests, safety evaluations, and regulatory standards of manufactured topical treatments is difficult. These multidisciplinary collaborations make it possible to better understand the role of the physicochemical properties of nanocarriers identified by material chemistry and processing for specific formulations in delivering therapeutic agents through the dermis. Integrating a rational design of nanocarriers with a wide-ranging benefit of such systems clinically can provide the basis for the most promising clinical trial approaches and potentially lead to real advancements. Clinical translation is primarily driven by clinical efficacy connected to enhanced biological effects of nanoformulations.

Consequently, more thorough research should be conducted to create nanobased medicine delivery for a commercial setting. The current review will serve as a building block for future research and significantly impact new product development and clinical results. Despite the fact that this review has shown the enormous potential of nanobased carriers, it is crucial to consider future developments in technology and strategies that enhance targeted topical delivery to fill in some of the gaps and overcome the difficulties topical delivery still faces. Numerous studies have already been conducted to show the therapeutic effectiveness of nanocarriers in the treatment and progression prevention of skin disorders. However, these drug delivery systems still need to be researched and developed to produce products that can be sold.

Additionally, more research is required to evaluate the therapeutic potential of nanocarriers in relevant clinical models of disease. In light of the aforementioned advantages, it is anticipated that new nanotechnology-based methodologies will be created which have the potential to revolutionize important facets of clinical dermatology. There is still much to learn about the topical application of nanotechnology as a therapy option for skin illnesses, even though extensive research is ongoing and significant discoveries have been made. Future research must continue the shift from preclinical investigations to clinical trials in order to have a more accurate understanding of the true impact of these nanocarriers. Finding the optimal nanocarrier for each type of skin disorder would be a fascinating future task that would help enhance nanotechnology’s advantages. Although more research is still needed, it is reasonable to claim that nanobased carriers provide a promising alternative to traditional therapy and improve safety and efficacy in the management of skin disorders.
